# 3β,5α,6β-Trihy­droxy­androstan-17-one

**DOI:** 10.1107/S1600536811011706

**Published:** 2011-04-07

**Authors:** L.C.R. Andrade, M.J.B.M. de Almeida, J.A. Paixão, J.F.S. Carvalho, M.L. Sá e Melo

**Affiliations:** a CEMDRX, Department of Physics, University of Coimbra, P-3004-516 Coimbra, Portugal; b Centre for Neuroscience and Cell Biology, University of Coimbra, P-3004-517 Coimbra, Portugal; c Faculty of Pharmacy, University of Coimbra, P-3000-548 Coimbra, Portugal

## Abstract

The title compound, C_19_H_30_O_4_, is an androstan-17-one derivative synthesized from the dehydro­epiandrosterone through a sequential addition of an oxidant, followed by a *trans*-diaxial opening of the epoxide generated, with Bi(OTf)_3_ (OTf is trifluoro­methane­sulfonate). The six-membered rings have a slightly flattened chair conformation, while the five-membered ring adopts a 14-α envelope conformation. All rings are *trans* fused. In the crystal, the mol­ecules are connected by O—H⋯O hydrogen bonds involving the hydroxyl and carbonyl groups, forming a three-dimensional network. A quantum mechanical *ab initio* Roothan Hartree–Fock calculation of the free mol­ecule gives bond lengths, valency angles and ring torsion angles of the free molecule at  equilibrium geometry (energy minimum) close to the experimental values.

## Related literature

For the synthesis of the title compound, see: Carvalho *et al.* (2010*b* ▶). For 3β,5α,6β-hy­droxy­lation pattern occurance in several natural products, see: Mizushina *et al.* (1999 ▶); Hata *et al.* (2002 ▶); Tanaka *et al.* (2002 ▶); Sun *et al.* (2006 ▶). For natural products as scaffolds for drug discovery, see: Li & Vederas (2009 ▶); Rosén *et al.* (2009 ▶). For angiotoxicity of 3β,5α,6β-trihy­droxy steroids, see: Imai *et al.* (1980 ▶); Peng *et al.* (1985 ▶). For the *in vivo* genesis of osteoporosis and atherosclerosis, see: Hongmei *et al.* (2005 ▶); Imai *et al.* (1980 ▶); Peng *et al.* (1985 ▶). For the cytotoxicity of steroids with a 3β,5α,6β-hy­droxy­lation motif against cancer cells, see: Aiello *et al.* (1995 ▶); Carvalho *et al.* (2010*a* ▶); El-Gamal *et al.* (2004 ▶). For the use of 3β,5α,6β-trihy­droxy steroids in the synthesis of Δ^4^-3,6-dione steroids. see: Tischler *et al.* (1988 ▶); Aiello *et al.* (1991 ▶); Pardo *et al.* (2000 ▶). For their use as mol­ecular probes for the study of aromatase inhibition, see: Numazawa & Tachibana (1994 ▶); Pérez-Ornelas *et al.* (2005 ▶); Nagaoka & Numazawa (2004 ▶). For the use of the title compound as an inter­mediate in the synthesis of the aromatase inhibitor androst-4-ene-3,6,17-trione, see: Ehrenstein (1939 ▶); Numazawa *et al.* (1987 ▶); Anthony *et al.* (1999 ▶). For related structures, see Anthony *et al.* (1999 ▶). For puckering parameters, see: Cremer & Pople (1975 ▶) and for asymmetry parameters, see: Duax & Norton (1975 ▶); Altona *et al.* (1968 ▶). For reference bond-length data, see: Allen *et al.* (1987 ▶). For the program *GAMESS* used to perform the quantum chemical calculations, see: Schmidt *et al.* (1993 ▶).
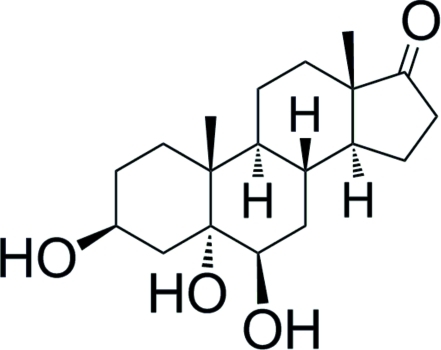


## Experimental

### 

#### Crystal data

C_19_H_30_O_4_
*M* *_r_* = 322.43
Orthorhombic, 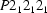

*a* = 5.8132 (1) Å
*b* = 13.3880 (3) Å
*c* = 21.3298 (5) Å
*V* = 1660.04 (6) Å^3^
*Z* = 4
Mo *K*α radiation
μ = 0.09 mm^−1^
*T* = 293 K
0.23 × 0.13 × 0.13 mm

#### Data collection

Bruker APEXII CCD area-detector diffractometer
Absorption correction: multi-scan (*SADABS*; Sheldrick, 2000 ▶) *T*_min_ = 0.937, *T*_max_ = 1.00
40718 measured reflections
2276 independent reflections
1874 reflections with *I* > 2σ(*I*)
*R*_int_ = 0.031

#### Refinement

*R*[*F* ^2^ > 2σ(*F* ^2^)] = 0.037
*wR*(*F* ^2^) = 0.098
*S* = 1.04
2276 reflections
213 parameters
H-atom parameters constrained
Δρ_max_ = 0.20 e Å^−3^
Δρ_min_ = −0.20 e Å^−3^

### 

Data collection: *APEX2* (Bruker, 2006 ▶); cell refinement: *SAINT* (Bruker, 2006 ▶); data reduction: *SAINT*; program(s) used to solve structure: *SHELXS97* (Sheldrick, 2008 ▶); program(s) used to refine structure: *SHELXL97* (Sheldrick, 2008 ▶); molecular graphics: *PLATON* (Spek, 2009 ▶); software used to prepare material for publication: *SHELXL97*.

## Supplementary Material

Crystal structure: contains datablocks global, I. DOI: 10.1107/S1600536811011706/bt5502sup1.cif

Structure factors: contains datablocks I. DOI: 10.1107/S1600536811011706/bt5502Isup2.hkl

Additional supplementary materials:  crystallographic information; 3D view; checkCIF report

## Figures and Tables

**Table 1:** Hydrogen-bond geometry (Å, °)

*D*—H⋯*A*	*D*—H	H⋯*A*	*D*⋯*A*	*D*—H⋯*A*
O3—H3⋯O17^i^	0.82	2.11	2.931 (2)	175
O5—H5⋯O3^ii^	0.82	1.99	2.8063 (19)	171
O6—H6*A*⋯O5^iii^	0.82	2.39	3.120 (2)	148

Symmetry codes: (i) 
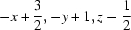
; (ii) 
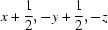
; (iii) 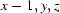.

**Fig. 1.**
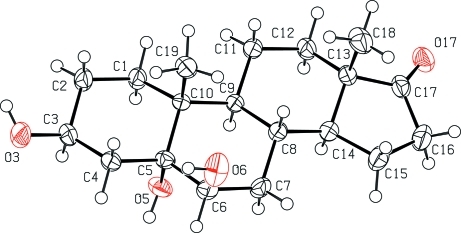
ORTEPII plot of the title compound. Displacement ellipsoids are drawn at the 50% level.

## supplementary crystallographic information

### Comment

Recently, the importance of natural products as scaffolds for drug discovery and design has been a subject of renewed interest (Li & Vederas, 2009; Rosén *et al.*, 2009). The 3β,5α,6β-hydroxylation pattern is found in several natural products (Mizushina *et al.*, 1999; Hata *et al.*, 2002; Tanaka *et al.*, 2002; Sun *et al.*, 2006) and also in human tissues, mainly in an oxidation product of cholesterol. The same hydroxylation motif is present in several natural steroids with interesting biological properties, namely cytotoxicity against cancer cells (Aiello *et al.*, 1995; El-Gamal *et al.*, 2004). On the other hand, cholestane-3β,5α,6β-triol has been extensively studied, proving to display cytotoxicity (Carvalho *et al.*, 2010*a*) and angiotoxicity (Imai *et al.*, 1980; Peng *et al.*, 1985) and has been suggested to participate in the *in vivo* genesis of pathological situations such as osteoporosis (Hongmei *et al.*, 2005) and atherosclerosis (Imai *et al.*, 1980; Peng *et al.*, 1985). Such findings validate the 3β,5α,6β-hydroxylation pattern as biologically important, and in this context a recently new protocol for the straightforward synthesis of 5α,6β-dihydroxy-steroids from a broad diversity of 3β-hydroxy-Δ^5^-steroids was accomplished by our group (Carvalho *et al.*, 2010*b*).

In addition, 3β,5α,6β-trihydroxy steroids are valuable intermediates for the synthesis of Δ^4^-3,6-dione-steroids, widely present in natural products (Tischler *et al.*, 1988; Aiello *et al.*, 1991; Pardo *et al.*, 2000) and with proved utility as molecular probes for the study of aromatase inhibition (Numazawa & Tachibana, 1994; Pérez-Ornelas *et al.*, 2005; Nagaoka & Numazawa, 2004). In fact, compound (I) is a synthetically valuable intermediate (Ehrenstein, 1939) of the biologically active androst-4-ene-3,6,17-trione compound, (Anthony *et al.*, 1999) which is a well known aromatase inhibitor (Numazawa *et al.*, 1987). Due to the interest of our group in the cytotoxic potential of steroids, a series of oxygenated steroids were further prepared and evaluated on HT-29 cancer cells (Carvalho *et al.*, 2010*a*). Compound (I) showed no relevant cytotoxicity (IC_50_ > 50µ*M*), in contrast to cholestane-3β,5α,6β-triol and other cholestane derivatives. Such result points to the importance of a C-17 cholesteryl type side chain for cytotoxicity thus the importance of X-ray difraction structural studies on such compounds.

Bond lengths and valency angles are within the range of expected values for this type of compounds (Allen *et al.*,1987) with the exception of bonds C2–C3 and C3–C4 [1.510 (3); 1.518 (3) Å)] which are significantely smaller than the C*sp*_3_–C*sp*_3_ average value [1.535 Å].

Rings A to C have slightly flattened chair conformations, as shown by the Cremer & Pople (1975) parameters [ring A: Q = 0.570 (2) Å, θ = 5.6 (2)° and φ = 299 (2)°; B: Q = 0.5705 (19) Å, θ = 3.4 (2)° and φ = 255 (3)°; C: Q = 0.5727 (19) Å, θ = 7.04 (19)° and φ = 271.3 (16)°].

Ring D has a 14-α envelope conformation [Cremer & Pople (1975) parameters q_2_ = 0.415 (2) Å and φ_2_ = 213.5 (3)° and asymmetry parameters (Duax & Norton, 1975; Altona *et al.*, 1968) ΔC_s_(14) =2.4 (2)°; ΔC_2_(13,14)=17.8 (2)°; φ_m_=42.6 (1)°; Δ=30.6 (4)°]. All rings are fused *trans*.

In order to gain some insight on how the crystal packing of (I) might affect the molecular geometry we have performed quantum chemical calculations on the equilibrium geometry of the free molecule. The calculations were performed with the computer program GAMESS (Schmidt *et al.*, 1993).

The *ab-initio* calculations reproduce well the observed experimental bond lengths and valency angles of the molecule. Also, the calculated conformation of the rings are very close to the experimental values.

The molecules are hydrogen-bonded *via* the hydroxyl and carbonyl groups forming a three-dimension hydrogen bond pattern. Each hydroxyl group acts as both donnor and acceptor, thus full potential for hydrogen bonding is achieved in the crystal struture. In addition to these bonds, three weak intramolecular interactions can be spotted involving atoms O5 and O6 and CH, CH_2_ and CH_3_ groups.

### Experimental

Synthesis of (I) was performed using a new and recently reported (Carvalho *et al.*, 2010*b*) fast and high yielding sequential chemical approach for the straightforward preparation of 5α,6β-dihydroxy-steroids using 3β-hydroxy-Δ^5^-steroids as raw materials. The protocol involves two steps: (i) formation of the epoxide from Δ^5^-steroids, using MMPP as oxidative agent; and (ii) *trans*-diaxial epoxide opening with Bi(OTf)_3_ in commercial acetone. Crystallization from ethanol at room temperature afforded colorless crystals suitable for X-ray analysis. Analytical data of compound (I) is in accordance with the literature (Carvalho *et al.*, 2010*b*).

### Refinement

All hydrogen atoms were refined as riding on their parent atoms using *SHELXL97* defaults. The absolute configuration was not determined from the X-ray data, as the molecule lacks any strong anomalous scatterer atom at the Mo Kα wavelength, but was known from the synthetic route. Friedel pairs were merged before refinement.

### Crystal data

**Table:**

C_19_H_30_O_4_	*D*_x_ = 1.290 Mg m^−^^3^
*M**_r_* = 322.43	Melting point: 574 K
Orthorhombic, *P*2_1_2_1_2_1_	Mo *K*α radiation, λ = 0.71073 Å
Hall symbol: P 2ac 2ab	Cell parameters from 6253 reflections
*a* = 5.8132 (1) Å	θ = 3.1–30.3°
*b* = 13.3880 (3) Å	µ = 0.09 mm^−^^1^
*c* = 21.3298 (5) Å	*T* = 293 K
*V* = 1660.04 (6) Å^3^	Prism, colourless
*Z* = 4	0.23 × 0.13 × 0.13 mm
*F*(000) = 704	

### Data collection

**Table:**

Bruker APEXII CCD area-detector diffractometer	2276 independent reflections
Radiation source: fine-focus sealed tube	1874 reflections with *I* > 2σ(*I*)
graphite	*R*_int_ = 0.031
φ and ω scans	θ_max_ = 27.9°, θ_min_ = 1.8°
Absorption correction: multi-scan (*SADABS*; Sheldrick, 2000)	*h* = −7→6
*T*_min_ = 0.937, *T*_max_ = 1.00	*k* = −17→17
40718 measured reflections	*l* = −27→25

### Refinement

**Table:**

Refinement on *F*^2^	Primary atom site location: structure-invariant direct methods
Least-squares matrix: full	Secondary atom site location: difference Fourier map
*R*[*F*^2^ > 2σ(*F*^2^)] = 0.037	Hydrogen site location: inferred from neighbouring sites
*wR*(*F*^2^) = 0.098	H-atom parameters constrained
*S* = 1.04	*w* = 1/[σ^2^(*F*_o_^2^) + (0.0563*P*)^2^ + 0.2093*P*] where *P* = (*F*_o_^2^ + 2*F*_c_^2^)/3
2276 reflections	(Δ/σ)_max_ < 0.001
213 parameters	Δρ_max_ = 0.20 e Å^−^^3^
0 restraints	Δρ_min_ = −0.20 e Å^−^^3^

### Special details

**Table:**

Experimental. IR (film) 3442, 3348, 2942, 2861, 1723, 1471, 1373, 1077, 1047, 1030, 1001, 960, 874 cm^-1^; ^1^H NMR (300 MHz, *DMSO-d6*) δ p.p.m. 0.77 (3*H*, s, 18-CH_3_), 1.04 (3*H*, s, 19-CH_3_), 2.36 (1*H*, dd, *J*=19.0, 8.2 Hz), 3.35 (1*H*, m, 6α-H), 3.74 (1*H*, s, OH), 3.78 (1*H*, m, 3α-H), 4.22 (1*H*, d, *J*=5.8 Hz, OH), 4.51 (1*H*, d, *J*=4.3 Hz, OH); ^13^C NMR (75 MHz, *DMSO-d6)*δ p.p.m. 13.4, 16.2, 20.0, 21.4 (CH_2_), 29.6, 31.0 (CH_2_), 31.5 (CH_2_), 32.0 (CH_2_), 33.3 (CH_2_), 35.3 (CH_2_), 37.9 (C-10), 40.8 (CH_2_), 44.8, 47.2 (C-13), 50.5, 65.6, 73.8, 74.3 (C-5), 220.0 (C-17); MS m/*z* (%): 321.3 (9) [M—H]^+^, 293.2 (20), 280.4 (23), 265.5 (100), 250.2 (13), 90.3 (54).
Geometry. All e.s.d.'s (except the e.s.d. in the dihedral angle between two l.s. planes) are estimated using the full covariance matrix. The cell e.s.d.'s are taken into account individually in the estimation of e.s.d.'s in distances, angles and torsion angles; correlations between e.s.d.'s in cell parameters are only used when they are defined by crystal symmetry. An approximate (isotropic) treatment of cell e.s.d.'s is used for estimating e.s.d.'s involving l.s. planes.
Refinement. Refinement of *F*^2^ against ALL reflections. The weighted *R*-factor *wR* and goodness of fit *S* are based on *F*^2^, conventional *R*-factors *R* are based on *F*, with *F* set to zero for negative *F*^2^. The threshold expression of *F*^2^ > σ(*F*^2^) is used only for calculating *R*-factors(gt) *etc*. and is not relevant to the choice of reflections for refinement. *R*-factors based on *F*^2^ are statistically about twice as large as those based on *F*, and *R*- factors based on ALL data will be even larger.

### Fractional atomic coordinates and isotropic or equivalent isotropic displacement parameters (Å^2^)

**Table:**

	*x*	*y*	*z*	*U*_iso_*/*U*_eq_	
O3	0.4447 (4)	0.39692 (11)	−0.03066 (6)	0.0551 (5)	
H3	0.4176	0.4562	−0.0371	0.083*	
O5	0.8749 (2)	0.26939 (10)	0.10844 (6)	0.0344 (3)	
H5	0.8788	0.2198	0.0860	0.052*	
O6	0.3172 (3)	0.20863 (13)	0.18197 (7)	0.0490 (4)	
H6A	0.2372	0.2182	0.1509	0.074*	
O17	1.1224 (3)	0.38845 (11)	0.44942 (6)	0.0396 (4)	
C1	0.7513 (4)	0.47124 (14)	0.11921 (8)	0.0304 (4)	
H1A	0.7555	0.5345	0.1414	0.036*	
H1B	0.9085	0.4527	0.1093	0.036*	
C2	0.6166 (4)	0.48453 (14)	0.05810 (8)	0.0358 (5)	
H2A	0.4642	0.5098	0.0676	0.043*	
H2B	0.6940	0.5334	0.0320	0.043*	
C3	0.5956 (4)	0.38742 (14)	0.02260 (8)	0.0334 (5)	
H3A	0.7485	0.3683	0.0075	0.040*	
C4	0.5032 (4)	0.30359 (13)	0.06337 (8)	0.0298 (4)	
H4A	0.5094	0.2414	0.0400	0.036*	
H4B	0.3433	0.3170	0.0733	0.036*	
C5	0.6392 (3)	0.29152 (13)	0.12470 (8)	0.0245 (4)	
C6	0.5535 (4)	0.20277 (14)	0.16369 (9)	0.0326 (5)	
H6	0.5743	0.1419	0.1388	0.039*	
C7	0.6922 (4)	0.19194 (13)	0.22384 (8)	0.0333 (5)	
H7A	0.8478	0.1718	0.2133	0.040*	
H7B	0.6247	0.1395	0.2492	0.040*	
C8	0.7015 (3)	0.28834 (13)	0.26229 (8)	0.0248 (4)	
H8	0.5454	0.3049	0.2762	0.030*	
C9	0.7942 (3)	0.37543 (13)	0.22212 (7)	0.0226 (4)	
H9	0.9469	0.3549	0.2076	0.027*	
C10	0.6459 (3)	0.39125 (12)	0.16226 (7)	0.0219 (4)	
C11	0.8298 (4)	0.47172 (13)	0.26035 (8)	0.0332 (5)	
H11A	0.9074	0.5205	0.2342	0.040*	
H11B	0.6805	0.4989	0.2714	0.040*	
C12	0.9702 (4)	0.45672 (14)	0.32058 (8)	0.0329 (5)	
H12A	1.1280	0.4402	0.3100	0.039*	
H12B	0.9712	0.5181	0.3448	0.039*	
C13	0.8657 (3)	0.37305 (14)	0.35939 (8)	0.0267 (4)	
C14	0.8548 (3)	0.27739 (13)	0.31973 (8)	0.0267 (4)	
H14	1.0109	0.2665	0.3038	0.032*	
C15	0.8117 (4)	0.19464 (15)	0.36767 (9)	0.0418 (5)	
H15A	0.8561	0.1299	0.3512	0.050*	
H15B	0.6513	0.1925	0.3801	0.050*	
C16	0.9667 (5)	0.22585 (15)	0.42264 (10)	0.0460 (6)	
H16A	0.8930	0.2110	0.4623	0.055*	
H16B	1.1126	0.1908	0.4209	0.055*	
C17	1.0028 (4)	0.33729 (15)	0.41549 (8)	0.0307 (4)	
C18	0.6314 (4)	0.40438 (18)	0.38719 (9)	0.0436 (5)	
H18A	0.6503	0.4641	0.4115	0.065*	
H18B	0.5739	0.3519	0.4135	0.065*	
H18C	0.5244	0.4166	0.3538	0.065*	
C19	0.4042 (3)	0.42658 (15)	0.18101 (9)	0.0331 (5)	
H19A	0.4118	0.4944	0.1955	0.050*	
H19B	0.3460	0.3847	0.2139	0.050*	
H19C	0.3038	0.4227	0.1454	0.050*	

### Atomic displacement parameters (Å^2^)

**Table:**

	*U*^11^	*U*^22^	*U*^33^	*U*^12^	*U*^13^	*U*^23^
O3	0.0995 (15)	0.0344 (8)	0.0314 (8)	−0.0083 (10)	−0.0306 (9)	−0.0014 (6)
O5	0.0335 (7)	0.0368 (8)	0.0328 (7)	0.0077 (6)	0.0005 (6)	−0.0113 (6)
O6	0.0397 (8)	0.0592 (10)	0.0482 (8)	−0.0219 (9)	−0.0106 (7)	0.0154 (8)
O17	0.0437 (8)	0.0463 (8)	0.0289 (7)	0.0028 (8)	−0.0082 (7)	−0.0058 (6)
C1	0.0425 (11)	0.0251 (9)	0.0235 (9)	−0.0085 (9)	−0.0074 (8)	0.0012 (7)
C2	0.0548 (13)	0.0278 (9)	0.0247 (9)	−0.0095 (10)	−0.0092 (10)	0.0023 (8)
C3	0.0461 (12)	0.0328 (10)	0.0213 (9)	−0.0015 (10)	−0.0054 (9)	−0.0025 (8)
C4	0.0398 (10)	0.0236 (9)	0.0260 (9)	−0.0050 (9)	−0.0069 (8)	−0.0041 (7)
C5	0.0256 (9)	0.0230 (9)	0.0250 (8)	−0.0013 (8)	−0.0021 (7)	−0.0039 (7)
C6	0.0417 (11)	0.0227 (9)	0.0335 (10)	−0.0083 (9)	−0.0086 (9)	−0.0010 (8)
C7	0.0468 (12)	0.0202 (9)	0.0330 (10)	−0.0055 (9)	−0.0082 (9)	0.0033 (7)
C8	0.0257 (9)	0.0237 (8)	0.0251 (8)	−0.0010 (8)	−0.0020 (7)	0.0016 (7)
C9	0.0249 (9)	0.0215 (8)	0.0213 (8)	−0.0010 (7)	−0.0017 (7)	−0.0010 (7)
C10	0.0249 (9)	0.0193 (8)	0.0216 (8)	−0.0004 (7)	−0.0009 (7)	−0.0018 (7)
C11	0.0504 (13)	0.0231 (9)	0.0262 (9)	−0.0037 (9)	−0.0092 (9)	−0.0002 (7)
C12	0.0458 (12)	0.0289 (9)	0.0240 (9)	−0.0060 (9)	−0.0066 (9)	−0.0018 (7)
C13	0.0293 (9)	0.0294 (9)	0.0215 (8)	0.0035 (8)	−0.0006 (8)	−0.0011 (7)
C14	0.0287 (10)	0.0256 (9)	0.0258 (8)	0.0003 (8)	−0.0008 (8)	0.0008 (7)
C15	0.0570 (14)	0.0325 (11)	0.0359 (10)	−0.0053 (11)	−0.0098 (10)	0.0095 (9)
C16	0.0661 (16)	0.0387 (11)	0.0332 (10)	0.0014 (12)	−0.0136 (11)	0.0086 (9)
C17	0.0310 (10)	0.0391 (11)	0.0220 (9)	0.0048 (9)	0.0027 (8)	−0.0005 (8)
C18	0.0364 (11)	0.0598 (14)	0.0346 (10)	0.0160 (11)	0.0025 (10)	−0.0060 (10)
C19	0.0303 (11)	0.0382 (11)	0.0306 (9)	0.0076 (9)	−0.0034 (9)	−0.0043 (8)

### Geometric parameters (Å, °)

**Table:**

O3—C3	1.441 (2)	C8—H8	0.9800
O3—H3	0.8200	C9—C11	1.539 (2)
O5—C5	1.444 (2)	C9—C10	1.555 (2)
O5—H5	0.8200	C9—H9	0.9800
O6—C6	1.430 (3)	C10—C19	1.535 (3)
O6—H6A	0.8200	C11—C12	1.535 (2)
O17—C17	1.215 (2)	C11—H11A	0.9700
C1—C2	1.531 (2)	C11—H11B	0.9700
C1—C10	1.538 (2)	C12—C13	1.520 (3)
C1—H1A	0.9700	C12—H12A	0.9700
C1—H1B	0.9700	C12—H12B	0.9700
C2—C3	1.510 (3)	C13—C17	1.515 (3)
C2—H2A	0.9700	C13—C14	1.536 (2)
C2—H2B	0.9700	C13—C18	1.543 (3)
C3—C4	1.518 (3)	C14—C15	1.528 (2)
C3—H3A	0.9800	C14—H14	0.9800
C4—C5	1.537 (2)	C15—C16	1.537 (3)
C4—H4A	0.9700	C15—H15A	0.9700
C4—H4B	0.9700	C15—H15B	0.9700
C5—C6	1.533 (3)	C16—C17	1.514 (3)
C5—C10	1.558 (2)	C16—H16A	0.9700
C6—C7	1.522 (3)	C16—H16B	0.9700
C6—H6	0.9800	C18—H18A	0.9600
C7—C8	1.530 (2)	C18—H18B	0.9600
C7—H7A	0.9700	C18—H18C	0.9600
C7—H7B	0.9700	C19—H19A	0.9600
C8—C14	1.522 (2)	C19—H19B	0.9600
C8—C9	1.544 (2)	C19—H19C	0.9600
			
C3—O3—H3	109.5	C19—C10—C1	107.80 (16)
C5—O5—H5	109.5	C19—C10—C9	109.59 (14)
C6—O6—H6A	109.5	C1—C10—C9	111.35 (14)
C2—C1—C10	112.67 (15)	C19—C10—C5	112.05 (14)
C2—C1—H1A	109.1	C1—C10—C5	107.44 (13)
C10—C1—H1A	109.1	C9—C10—C5	108.61 (13)
C2—C1—H1B	109.1	C12—C11—C9	113.91 (15)
C10—C1—H1B	109.1	C12—C11—H11A	108.8
H1A—C1—H1B	107.8	C9—C11—H11A	108.8
C3—C2—C1	111.62 (15)	C12—C11—H11B	108.8
C3—C2—H2A	109.3	C9—C11—H11B	108.8
C1—C2—H2A	109.3	H11A—C11—H11B	107.7
C3—C2—H2B	109.3	C13—C12—C11	109.85 (16)
C1—C2—H2B	109.3	C13—C12—H12A	109.7
H2A—C2—H2B	108.0	C11—C12—H12A	109.7
O3—C3—C2	111.65 (16)	C13—C12—H12B	109.7
O3—C3—C4	107.56 (16)	C11—C12—H12B	109.7
C2—C3—C4	112.23 (14)	H12A—C12—H12B	108.2
O3—C3—H3A	108.4	C17—C13—C12	116.91 (17)
C2—C3—H3A	108.4	C17—C13—C14	101.13 (14)
C4—C3—H3A	108.4	C12—C13—C14	109.33 (14)
C3—C4—C5	112.54 (15)	C17—C13—C18	104.28 (15)
C3—C4—H4A	109.1	C12—C13—C18	111.22 (17)
C5—C4—H4A	109.1	C14—C13—C18	113.69 (16)
C3—C4—H4B	109.1	C8—C14—C15	120.83 (16)
C5—C4—H4B	109.1	C8—C14—C13	112.78 (14)
H4A—C4—H4B	107.8	C15—C14—C13	104.05 (14)
O5—C5—C6	106.23 (15)	C8—C14—H14	106.1
O5—C5—C4	107.77 (14)	C15—C14—H14	106.1
C6—C5—C4	112.08 (14)	C13—C14—H14	106.1
O5—C5—C10	106.01 (13)	C14—C15—C16	102.55 (16)
C6—C5—C10	113.17 (13)	C14—C15—H15A	111.3
C4—C5—C10	111.12 (14)	C16—C15—H15A	111.3
O6—C6—C7	106.51 (16)	C14—C15—H15B	111.3
O6—C6—C5	114.67 (17)	C16—C15—H15B	111.3
C7—C6—C5	111.02 (15)	H15A—C15—H15B	109.2
O6—C6—H6	108.1	C17—C16—C15	105.81 (17)
C7—C6—H6	108.1	C17—C16—H16A	110.6
C5—C6—H6	108.1	C15—C16—H16A	110.6
C6—C7—C8	112.96 (15)	C17—C16—H16B	110.6
C6—C7—H7A	109.0	C15—C16—H16B	110.6
C8—C7—H7A	109.0	H16A—C16—H16B	108.7
C6—C7—H7B	109.0	O17—C17—C16	125.09 (19)
C8—C7—H7B	109.0	O17—C17—C13	126.38 (17)
H7A—C7—H7B	107.8	C16—C17—C13	108.53 (17)
C14—C8—C7	111.77 (14)	C13—C18—H18A	109.5
C14—C8—C9	108.37 (14)	C13—C18—H18B	109.5
C7—C8—C9	110.60 (14)	H18A—C18—H18B	109.5
C14—C8—H8	108.7	C13—C18—H18C	109.5
C7—C8—H8	108.7	H18A—C18—H18C	109.5
C9—C8—H8	108.7	H18B—C18—H18C	109.5
C11—C9—C8	112.67 (13)	C10—C19—H19A	109.5
C11—C9—C10	113.30 (14)	C10—C19—H19B	109.5
C8—C9—C10	111.41 (14)	H19A—C19—H19B	109.5
C11—C9—H9	106.3	C10—C19—H19C	109.5
C8—C9—H9	106.3	H19A—C19—H19C	109.5
C10—C9—H9	106.3	H19B—C19—H19C	109.5
			
C10—C1—C2—C3	−56.4 (2)	O5—C5—C10—C1	59.79 (17)
C1—C2—C3—O3	172.65 (17)	C6—C5—C10—C1	175.84 (15)
C1—C2—C3—C4	51.8 (2)	C4—C5—C10—C1	−57.03 (19)
O3—C3—C4—C5	−175.47 (15)	O5—C5—C10—C9	−60.77 (17)
C2—C3—C4—C5	−52.3 (2)	C6—C5—C10—C9	55.28 (19)
C3—C4—C5—O5	−60.09 (19)	C4—C5—C10—C9	−177.60 (14)
C3—C4—C5—C6	−176.63 (16)	C8—C9—C11—C12	50.7 (2)
C3—C4—C5—C10	55.7 (2)	C10—C9—C11—C12	178.37 (16)
O5—C5—C6—O6	−177.18 (15)	C9—C11—C12—C13	−52.8 (2)
C4—C5—C6—O6	−59.7 (2)	C11—C12—C13—C17	170.85 (16)
C10—C5—C6—O6	66.9 (2)	C11—C12—C13—C14	56.8 (2)
O5—C5—C6—C7	62.06 (18)	C11—C12—C13—C18	−69.57 (19)
C4—C5—C6—C7	179.52 (15)	C7—C8—C14—C15	−55.8 (2)
C10—C5—C6—C7	−53.9 (2)	C9—C8—C14—C15	−177.90 (16)
O6—C6—C7—C8	−72.2 (2)	C7—C8—C14—C13	−179.62 (16)
C5—C6—C7—C8	53.2 (2)	C9—C8—C14—C13	58.24 (19)
C6—C7—C8—C14	−176.03 (16)	C17—C13—C14—C8	173.72 (15)
C6—C7—C8—C9	−55.2 (2)	C12—C13—C14—C8	−62.4 (2)
C14—C8—C9—C11	−51.4 (2)	C18—C13—C14—C8	62.6 (2)
C7—C8—C9—C11	−174.26 (16)	C17—C13—C14—C15	41.03 (18)
C14—C8—C9—C10	179.96 (14)	C12—C13—C14—C15	164.95 (17)
C7—C8—C9—C10	57.1 (2)	C18—C13—C14—C15	−70.1 (2)
C2—C1—C10—C19	−62.94 (19)	C8—C14—C15—C16	−168.04 (17)
C2—C1—C10—C9	176.82 (15)	C13—C14—C15—C16	−40.2 (2)
C2—C1—C10—C5	58.0 (2)	C14—C15—C16—C17	23.2 (2)
C11—C9—C10—C19	−62.1 (2)	C15—C16—C17—O17	−177.7 (2)
C8—C9—C10—C19	66.23 (18)	C15—C16—C17—C13	2.2 (2)
C11—C9—C10—C1	57.1 (2)	C12—C13—C17—O17	34.9 (3)
C8—C9—C10—C1	−174.59 (14)	C14—C13—C17—O17	153.5 (2)
C11—C9—C10—C5	175.23 (15)	C18—C13—C17—O17	−88.3 (2)
C8—C9—C10—C5	−56.48 (18)	C12—C13—C17—C16	−145.00 (19)
O5—C5—C10—C19	178.02 (15)	C14—C13—C17—C16	−26.4 (2)
C6—C5—C10—C19	−65.9 (2)	C18—C13—C17—C16	91.8 (2)
C4—C5—C10—C19	61.19 (19)	C19—C10—C13—C18	1.68 (16)

### Hydrogen-bond geometry (Å, °)

**Table:**

*D*—H···*A*	*D*—H	H···*A*	*D*···*A*	*D*—H···*A*
O3—H3···O17^i^	0.82	2.11	2.931 (2)	175.
O5—H5···O3^ii^	0.82	1.99	2.8063 (19)	171.
O6—H6A···O5^iii^	0.82	2.39	3.120 (2)	148.

Symmetry codes: (i) −*x*+3/2, −*y*+1, *z*−1/2; (ii) *x*+1/2, −*y*+1/2, −*z*; (iii) *x*−1, *y*, *z*.
